# Luteoloside Suppresses Proliferation and Metastasis of Hepatocellular Carcinoma Cells by Inhibition of NLRP3 Inflammasome

**DOI:** 10.1371/journal.pone.0089961

**Published:** 2014-02-26

**Authors:** Shao-hua Fan, Yan-yan Wang, Jun Lu, Yuan-lin Zheng, Dong-mei Wu, Meng-qiu Li, Bin Hu, Zi-feng Zhang, Wei Cheng, Qun Shan

**Affiliations:** 1 Key Laboratory for Biotechnology on Medicinal Plants of Jiangsu Province, School of Life Science, Jiangsu Normal University, Xuzhou, Jiangsu, China; 2 Department of Function Examination, The First People's Hospital of Xuzhou, Jiangsu, China; 3 School of Environment and Spatial Informatics, China University of Mining and Technology, Xuzhou, Jiangsu, China; Virginia Commonwealth University, United States of America

## Abstract

The inflammasome is a multi-protein complex which when activated regulates caspase-1 activation and IL-1β secretion. Inflammasome activation is mediated by NLR proteins that respond to stimuli. Among NLRs, NLRP3 senses the widest array of stimuli. NLRP3 inflammasome plays an important role in the development of many cancer types. However, Whether NLRP3 inflammasome plays an important role in the process of hepatocellular carcinoma (HCC) is still unknown. Here, the anticancer effect of luteoloside, a naturally occurring flavonoid isolated from the medicinal plant *Gentiana macrophylla*, against HCC cells and the underlying mechanisms were investigated. Luteoloside significantly inhibited the proliferation of HCC cells *in vitro* and *in vivo*. Live-cell imaging and transwell assays showed that the migration and invasive capacities of HCC cells, which were treated with luteoloside, were significantly inhibited compared with the control cells. The inhibitory effect of luteoloside on metastasis was also observed *in vivo* in male BALB/c-nu/nu mouse lung metastasis model. Further studies showed that luteoloside could significantly reduce the intracellular reactive oxygen species (ROS) accumulation. The decreased levels of ROS induced by luteoloside was accompanied by decrease in expression of NLRP3 inflammasome resulting in decrease in proteolytic cleavage of caspase-1. Inactivation of caspase-1 by luteoloside resulted in inhibition of IL-1β. Thus, luteoloside exerts its inhibitory effect on proliferation, invasion and metastasis of HCC cells through inhibition of NLRP3 inflammasome. Our results indicate that luteoloside can be a potential therapeutic agent not only as an adjuvant therapy for HCC, but also, in the control and prevention of metastatic HCC.

## Introduction

Hepatocellular carcinoma (HCC) is the third leading cause of cancer-induced death worldwide, and patients have a very poor prognosis [1], [2]. Usually, HCC is treated by surgical resection or liver transplantation, which curative options for the patients when the disease is diagnosed at an early stage. However, approximately 70% of patients are inoperable because of tumor metastasis [3]. The current therapeutic options for HCC are not very effective because it is resistant to chemotherapy. Furthermore, many anti-cancer drugs have toxicity and side effects for the patients. Thus novel therapeutic strategies are needed to decrease the incidence and severity associated with this cancer [4]. Therefore, there is a pressing need for new therapeutic drugs with increased efficacy and decreased toxicity.

Natural products continue to provide promising lead compounds and drug candidates in modern antitumor drug discovery. Flavonoids are a heterogeneous group of polyphenolic compounds found ubiquitously in a wide variety of plants. Our recent reports show that they display a wide range of pharmacological properties, e.g., anti-inflammatory and antioxidative activities [5], [6]. The anti-tumor activity of flavonoid has recently attracted much attention [7]–[9]. Luteoloside (luteolin-7-*O*-glucoside; cynaroside; CAS 5373-11-5), a flavone subclass of flavonoids, possesses potential anti-inflammatory [10], free radical scavenging [11] and antibacterial [12]. Although it is reported that luteoloside could inhibit the proliferation of colon cancer cells [13], the exact mechanism remains unclear. Furthermore, the precise impact of luteoloside on cancer migration and invasion is still unreported.

NLR family, pyrin domain containing 3 (NLRP3; also known as NALP3 or cryopyrin) is a member of the nucleotide-binding domain and leucine-rich repeat containing gene family of intracellular sensors. When activated, NLRP3 forms a protein complex called the inflammasome [14]. The inflammasome combines NLRP3 with the adaptor molecule ASC/PYCARD/TMS/CARD5, Cardinal, and pro-caspase-1 to form a multimer. The result is the proteolytic maturation of caspase-1, which cleaves and activates proIL-1β to mature and active IL-1β [14]–[16]. NLRP3 inflammasome plays an important role in the development of many cancer types, including melanoma [17], intestinal cancer [18], nasopharyngeal carcinoma [19], skin cancer [20], colorectal cancer [21]. However, Whether NLRP3 inflammasome plays an important role in the process of HCC, to our knowledge, is still unknown.

In the present study, we demonstrate that luteoloside is a potent agent against human hepatoma cells both *in vitro* and *in vivo*, and NLRP3 inflammasome might be involved in the signaling of luteoloside-induced suppression of proliferation, migration and invasion. Our data provide the mechanistic insight into the role of luteoloside in inhibition of HCC cell proliferation, migration and invasion.

## Materials and Methods

### Cell Lines and Reagents

The human HCC cell lines (Hep3B and SNU-449) were purchased from the American Type Culture Collection. Human hepatoma cells (Huh-7) was purchased from Japanese Collection of Research Bioresources (JCRB, Tokyo, Japan). The human hepatoma cell line SMMC-7721 was purchased from the Committee on Type Culture Collection of Chinese Academy of Sciences (Shanghai, China). The human HCC cell lines (MHCC-LM3 and MHCC97-H) were obtained from the Liver Cancer Institute of Zhongshan Hospital, Fudan University (Shanghai, China).

**Figure 1:**
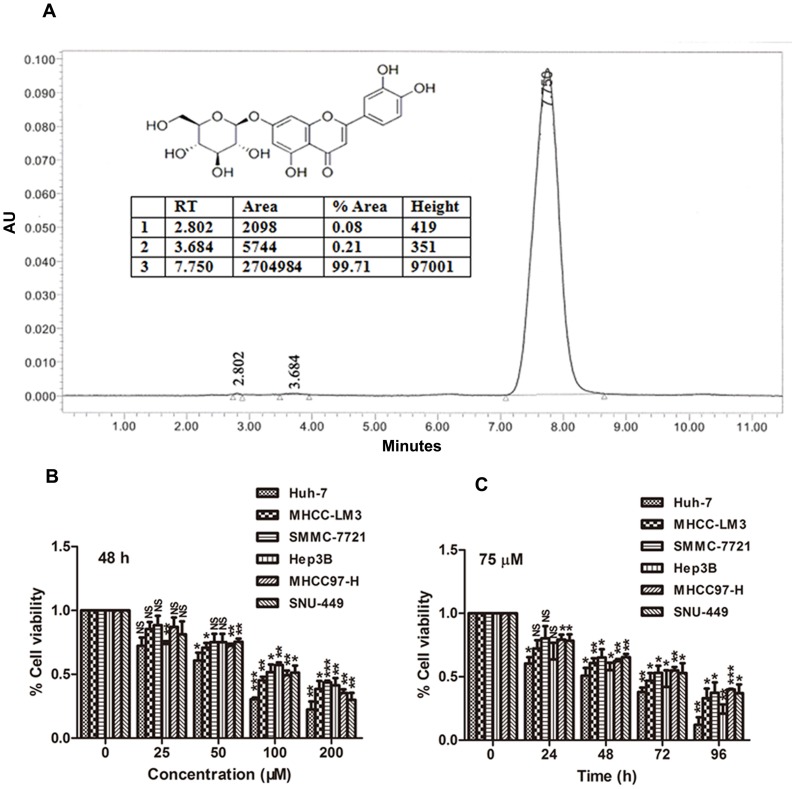
Luteoloside inhibits proliferation of HCC cells. (A) HPLC analysis of the purity of luteoloside used in the present study. *Insert*: The chemical structure of luteoloside. (B)–(C), comparative dose- and time-dependent effect of luteoloside on the proliferation potential of HCC cells. The percentage of cell viability in different treatment groups was determined using Cell Counting Kit-8 assay. *, *P*<0.05; **, *P*<0.01; ***, *P*<0.001; NS, not significant (*P*>0.05), versus non-luteoloside-treated control group.

Luteoloside (Fig. 1A, Batch Number: 025-120622, Purity = 99.7%, purchased from Chengdu Herbpurify Co., Ltd., Chengdu, China), a naturally occurring flavonoid isolated from the medicinal plant *Gentiana macrophylla*, was dissolved at a concentration of 20 mM in 100% DMSO as a stock solution, stored at −20°C, and diluted with medium before each experiment. The final DMSO concentration did not exceed 0.1% throughout the study.

### Caspase-3/7 Activity Assay

The caspase-3/7 activity assay was conducted as previously described by us [22].

### 2.3. DNA Fragmentation Assay

DNA from Huh-7 and SMMC-7721 cells (2×10^6^ cells) treated with 0 or 50 µM luteoloside for 24 hours was extracted by using a DNA extraction kit (Beyotime, China). The DNA (1500-ng aliquots) was resolved by electrophoresis on a 1.5% agarose gel containing 0.5 µg/mL ethidium bromide and was visualized under ultraviolet light [23].

### ROS Assay

The generation of ROS was assessed in Huh-7 or SMMC-7721 cells with the 2′,7′-dichlorofluorescein diacetate (DCFH-DA) (Invitrogen) probe, which is hydrolyzed within cells to non-fluorescent 2′,7′-dichlorodihydrofluorescin (DCFH). DCFH can be oxidized to the fluorescent 2′,7′-dichlorofluorescein (DCF) by hydroxyl radicals, peroxynitrite, and nitric oxide. Briefly, Huh-7 or SMMC-7721 cells were seeded in a 96-well plate. Overnight, the cells were incubated with different concentration of luteoloside for 8 h, then reacted with 10 µM DCFH-DA at 37°C for 20 min. Or the cells were incubated with NAC (10 mM), H_2_O_2_ (100 µM), diamide (10 mM) or BSO (100 µM) for 4 h, followed by 50 µM luteoloside for 4 h [24]. DCF was determined at λ_ex_ = 490 and λ_em_ = 520 nm on a Synergy H4 microplate reader (BioTek, Winooski, VT). Furthermore, ROS were measured with a Leica DMI4000B inverted fluorescence (Leica, Wetzlar, Germany).

### Protein Extraction and Western Blotting

The proteins were separated by SDS-PAGE and transferred to nitrocellulose membrane (Bio-Rad, Hercules, CA). The membrane was blocked with 5% non-fat milk and incubated with rabbit anti-LC3 polyclonal antibody (pAb) (Novus Biologicals) (2 µg/ml), rabbit anti-Beclin 1 pAb (Abcam) (3 µg/ml), rabbit anti-NLRP3 pAb (Novus Biologicals) (1∶1000), rabbit anti-caspase-1 (p10) pAb (Santa Cruz Biotechnology) (1∶1000), rabbit anti-IL-1β pAb (Santa Cruz Biotechnology) (1∶1000) or rabbit anti-β-actin pAb (Bioworld Technology) (1∶5000). The proteins were detected with enhanced chemiluminescence reagents (Pierce).

### Cell Proliferation Assay

The cell proliferation assay was conducted as previously described by us [22].

### Scratch-wound Assay

Scratch-wound assay was conducted as previously described by us [22]. The migration of cells into the wound was monitored in multiple wells using a CellVoyager CV1000 confocal scanner system (Yokogawa Electronic, Tokyo, Japan) with an Olympus UPLSApo 10×2 10×/0.4 Dry ∞/0.17/26.5 WD 3.1 plan super apochromat objective lens. The images were acquired every 0.5 hour for 48 hours (or every hour for 72 hours). The images shown represent 0 and 48 hour (or 0 and 72 hour).

### *In Vitro* Migration and Invasion Assays

Assays were performed as described previously by Yao *et al* [25].

### Xenograft Model and Treatments

Two different mouse models were used to observe *in vivo* effect of Luteoloside on HCC cells. For the subcutaneous model, the mice (male BALB/c-nu/nu, 6 weeks old) were anesthetized using 1% sodium pentobarbital (0.2 ml/20 g body weight, Sigma Chemical), as described by us previously [22]. The SMMC-7721 cells (2×10^6^ cells) were suspended in 200 µl serum-free DMEM and subcutaneously injected into the right upper flank of each mouse. Two weeks after the cells were injected, when tumors were observable, the animals were equally divided into two groups (ten per group). The first group received only 0.2 ml of vehicle material by gavage daily and served as a control group. The second group of animals received luteoloside (2 mg/kg body weight; equivalent to a dose of 6.5 mg/m^2^ in patients) in vehicle, respectively, for 4 weeks. Body weight was measured every 4 days to adjust the drug dosage. The tumors were measured using digital calipers every 3 to 4 days after they reached a volume of 100 mm^3^, and tumor volumes were calculated as described: V (cm^3^) = Width^2^ (cm^2^)×Length (cm)/2. At the termination of the experiment, the mice were sacrificed by cervical dislocation, and the tumors were weighed immediately after dissection.

For lung metastasis experiments, 1×10^6^ SMMC-7721 cells were suspended in 100 µl PBS and injected into the tail veins of each mouse (male BALB/c-nu/nu, 6 weeks old) [26]. Then, the animals were equally divided into two groups (ten per group). The first group received only 0.2 ml of vehicle material by gavage daily and served as a control group. The second group of animals received Luteoloside (2 mg/kg body weight) in vehicle, respectively, for 8 weeks. Body weight was measured every 4 days to adjust the drug dosage. At the termination of the experiment, the mice were sacrificed by cervical dislocation, and their lungs were removed and subjected to hematoxylin & eosin (H&E) staining.

This study was carried out in strict accordance with the recommendations in the Guide for the Care and Use of Laboratory Animals of the National Institutes of Health. The protocol was approved by the Committee on the Ethics of Animal Experiments of the University of Jiangsu Normal University (Permit Number: 13-0221). All surgery was performed under sodium pentobarbital anesthesia, and all efforts were made to minimize suffering.

### Statistical Analysis

Data are presented as means ± SEM and comparisons were made using Student’s *t* test. A probability of 0.05 or less was considered statistically significant.

## Results

### Luteoloside Inhibits the Proliferation of HCC Cells *in vitro*

We first determined whether luteoloside inhibits the proliferation of human HCC cells. We found that luteoloside significantly inhibited cell proliferation in all six-cell lines in a dose- and time-dependent manner (Fig. 1B, 1C). The results suggest that luteoloside has promising antihepatoma activity.

### Luteoloside Inhibits the Migration and Invasion of HCC Cells *in vitro*

**Figure 2:**
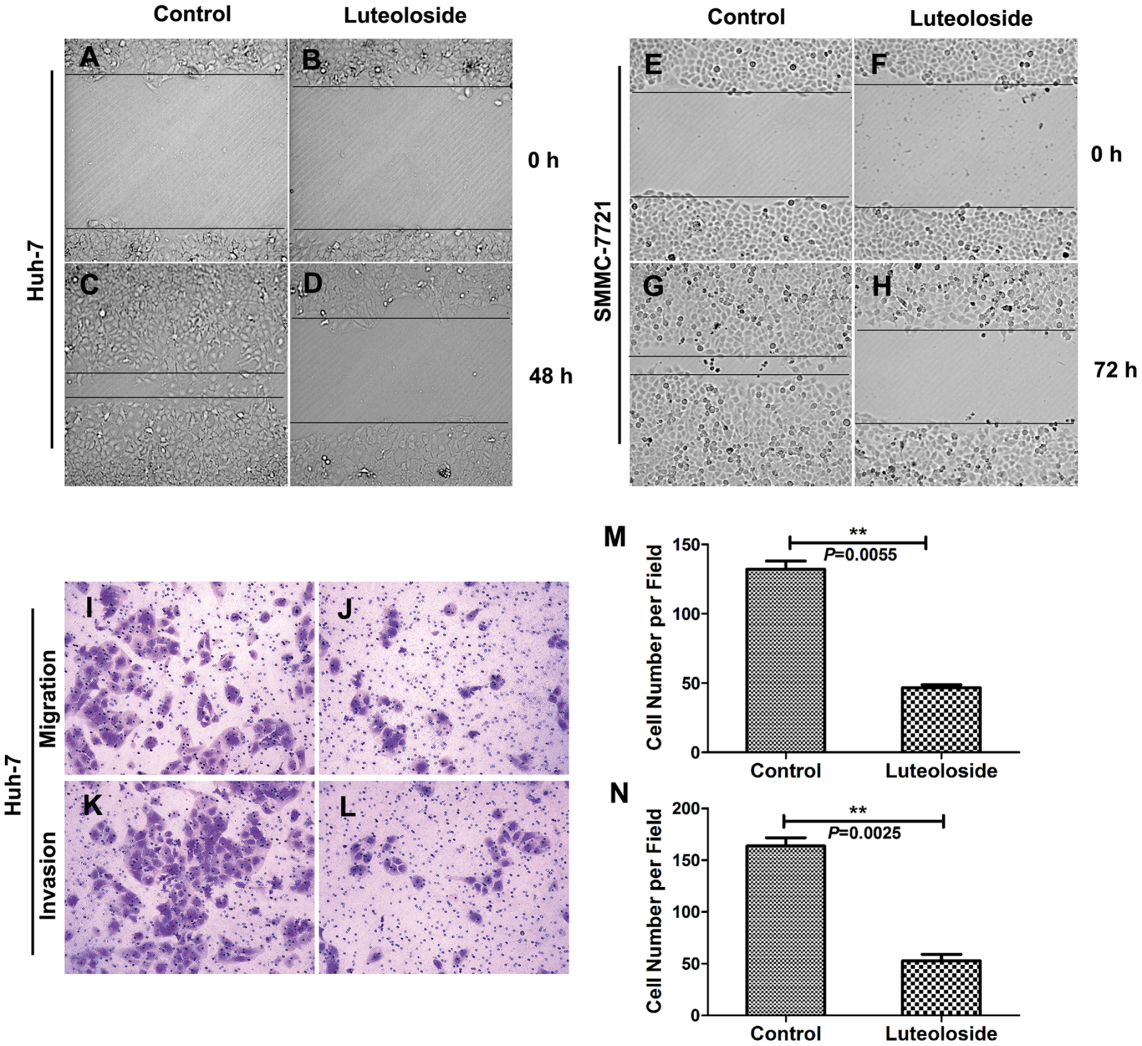
Luteoloside inhibits migration and invasion of HCC cells. The migration of cells into the wound was monitored in multiple wells using a CellVoyager CV1000 confocal scanner system. The images were acquired every 0.5 hour for 48 hours (Huh-7 cells) or every hour for 72 hours (SMMC-7721 cells) (see Supplemental Movies, 1–4). The images shown represent 0 hour (A, B, E, F), 48 hours (C, D) and 72 hours (G, H). The distance between the two edges of the scratch in the luteoloside-treated cells (D or H) was greater than that of the control (C or G). (I–N) Transwell migration and invasion assays of Huh-7 cells. For the transwell migration assay, 5×10^4^ cells were placed on the top chamber of each insert with the noncoated membrane. For the invasion assay, 1×10^5^ cells were placed on the upper chamber of each insert coated with 150 µg Matrigel (BD Biosciences, MA). Cells in both assays were trypsinized and resuspended in DMEM, and 700–900 µL of medium supplemented with 10% fetal bovine serum was injected into the lower chambers. Representative images are shown on the left (I, J, K, L), and the quantification of five randomly selected fields is shown on the right (M, N). The values shown are expressed as the mean ± SEM. **, *P*<0.01, versus non-luteoloside-treated control group. Scale bar: 100 µm.

**Figure 3:**
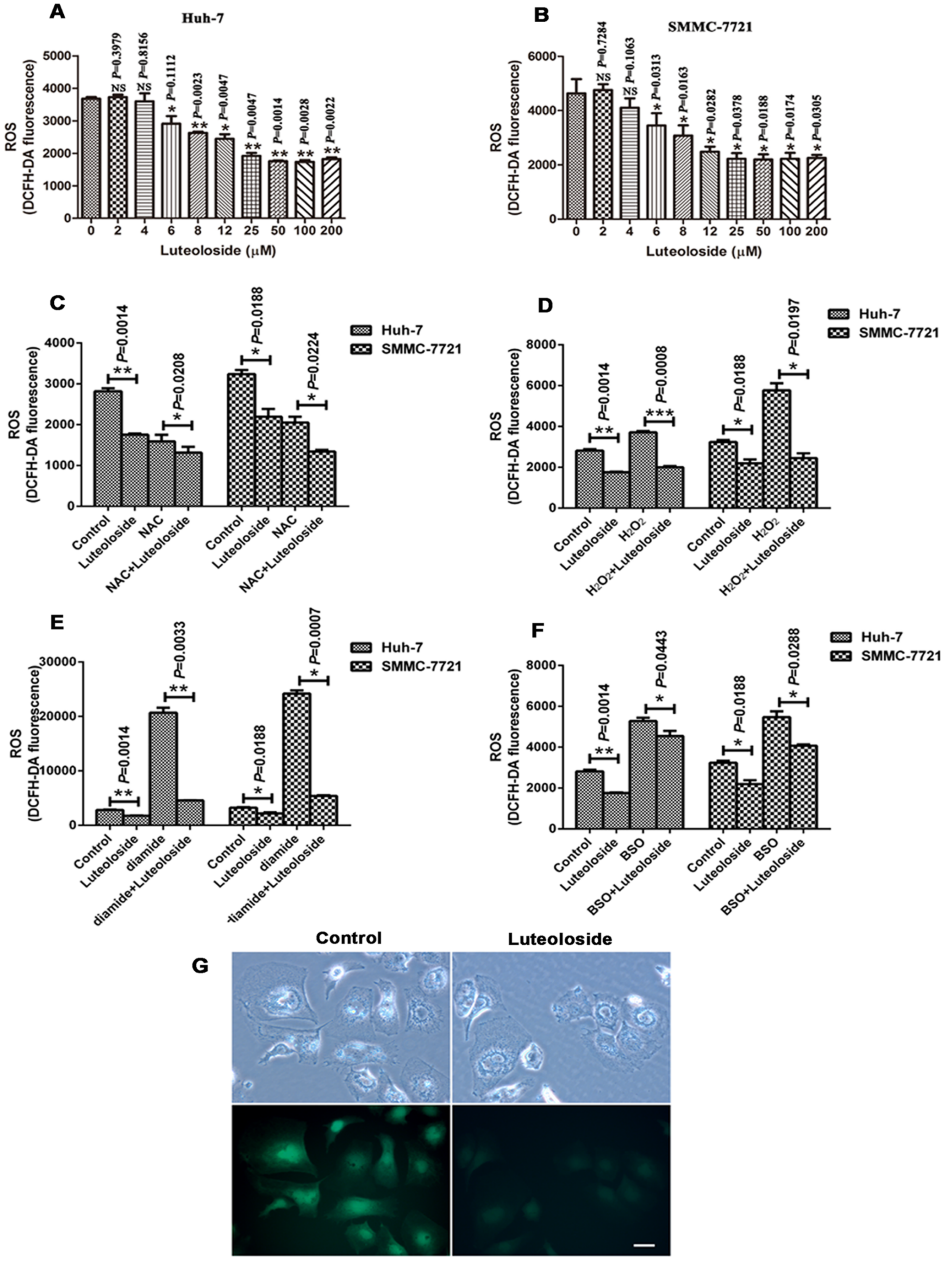
Luteoloside decreases intracellular ROS. ROS levels were measured using the ROS assay with DCFH-DA fluorescence dye. (A–B) Cells were treated with luteoloside at the indicated concentration for 8 h, then reacted with 10 µM DCFH-DA for 20 min. DCF fluorescence was determined on a Synergy H4 microplate reader. Cells were incubated with NAC (C), H_2_O_2_ (D), diamide (E) or BSO (F) for 4 h, followed by 50 µM luteoloside for 4 h. DCF was determined on a microplate reader. (G) DCFH-DA fluorescence (green) imaging of ROS in Huh-7 cells. Scale bar: 25 µm.

Luteoloside significantly decreased the migration of Huh-7 and SMMC-7721 cells compared with the control groups (Fig. 2A–2H; Supplementary Movies 1–4). Transwell assays without Matrigel demonstrated that luteoloside could significantly inhibit migration of Huh-7 cells when compared with control groups (Fig. 2I, 2J, 2M). Transwell assays with Matrigel showed that the invasive capacities of Huh-7 cells, which were treated with luteoloside, were significantly inhibited compared with the control cells (Fig. 3K, 3L, 3N). These results indicate that luteoloside can significantly inhibit HCC cells migration and invasion *in vitro*.

### Luteoloside has no Apoptotic Effects on HCC Cells

Huh-7 and SMMC-7721 cells were treated with luteoloside for 24 h, and caspase-3/7 was measured. The results showed that caspase-3/7 activity was not significantly different between luteoloside-treated cells and control cells when added 5, 10, 20, 50, 100, 150 or 200 µM luteoloside, respectively (Fig. S1A, S1B). Similar results were obtained by analyzing changes in nuclear fragmentation (Fig. S1C) and condensation (Fig. S1D) in cells. These results indicated that luteoloside has no apoptotic effects on Huh-7 and SMMC-7721 cells.

### Luteoloside does not Affect Autophagy

Autophagic cell death (also known as Type II programmed cell death to distinguish it from apoptosis or Type I programmed cell death) has been described as a distinct form of cell death that differs from other death mechanism such as apoptosis and necrosis. Next, we investigated whether luteoloside can induce autophagy in HCC cells. Beclin 1 and LC3 (microtubule-associated protein 1A/1B-light chain 3) play a pivotal role in mammalian autophagy. Beclin 1 is involved in both the signaling pathway activating autophagy and in the initial step of autophagosome formation [27]. LC3 comprises both a soluble LC3-I and a lapidated form, called LC3-II. LC3-II correlates with autophagy, being recruited into autophagosomes. Various types of stressors up-regulate LC3 and promote the conjugation of its cytosolic form, LC3-I to phosphatidylethanolamine, to constitute the autophagosome-specific LC3-II, which is so far considered the most reliable marker of autophagy [27], [28]. Huh-7 and SMMC-7721 cells were treated with luteoloside for 48 h, and the levels of LC3 and Beclin 1 proteins of different treatment groups were determined. The results showed that LC3 protein level was not significantly different between luteoloside-treated cells and control cells when added 25 µM or 50 µM luteoloside, respectively. Similar results were obtained by analyzing changes in levels of Beclin 1 (Fig. S2). These results indicated that luteoloside has no autophagic effects on Huh-7 and SMMC-7721 cells.

### Luteoloside Reduces Intracellular ROS Accumulation

ROS and cellular oxidant stress have long been associated with cancer [29]. Flavonoids are well known as ROS scavengers. As luteoloside is a kind of flavonoid isolated from Chinese herb [30], we investigated whether the intracellular ROS is part of the mechanism by which luteoloside suppress the proliferation, migration and invasion potential of HCC cells. We found that luteoloside could significantly decrease the ROS level of Huh-7 and SMMC-7721 cells in a dose-dependent manner (Fig. 3A, 3B). *N*-acetyl-cysteine (NAC) is a ROS-specific inhibitor [31]. NAC was shown to be capable of suppressing the ROS production in Huh-7 and SMMC-7721 cells (Fig. 3C). When the cells were pretreated with 10 mM NAC for 4 h, then treated with 50 µM luteoloside for 4 h, the ROS level was significantly lower than the cells which treated with 10 mM NAC only (Huh-7 cells, *P* = 0.0208; SMMC-7721 cell, *P* = 0.0224). H_2_O_2_, diamide and BSO are all ROS inducers [4]. Treatment with 100 µM H_2_O_2_, 10 mM diamide or 100 µM BSO showed similar effects, resulted in an increase in ROS levels, compared with control (Fig. 3D–3F). The results showed that H_2_O_2_, diamide and BSO could significantly increase the ROS level of Huh-7 and SMMC-7721 cells compared the control group (Fig. 3D–3F). However, after a prolonged time, when the cells were treated with 50 µM luteoloside for 4 h, the amount of ROS could significantly decrease (Fig. 3D–3F). Furthermore, the ROS in Huh-7 cells were monitored using a fluorescence microscope. We also found that luteoloside could significantly decrease the ROS level of Huh-7 cells (Fig. 3G).

### Luteoloside Downregulates the Expression Level of NLRP3, Caspase-1 (p10) and IL-1β

**Figure 4:**
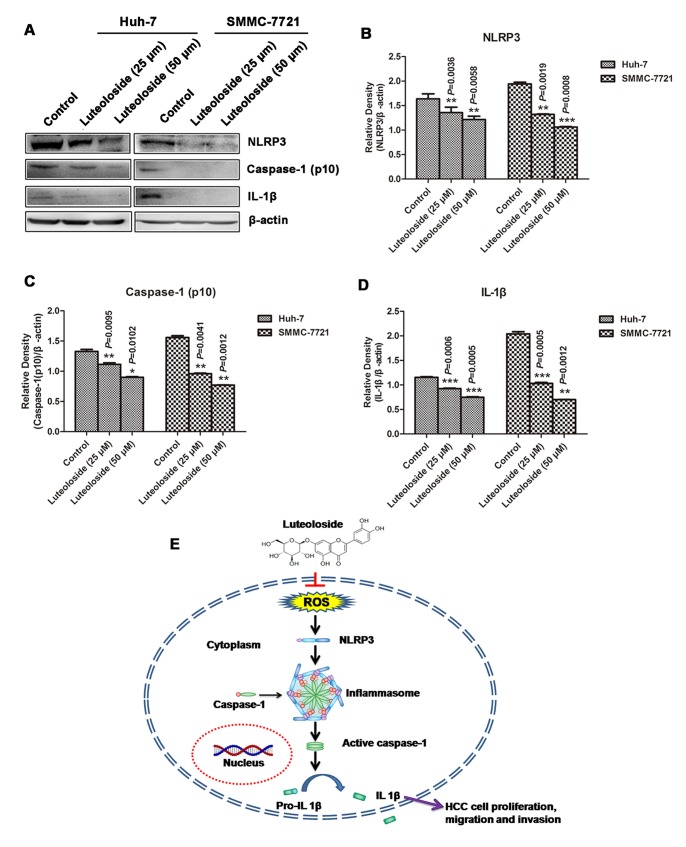
Luteoloside suppresses the NLRP3 inflammasome activation. (A) Western blot analyses of NLRP3, Caspase-1 (p10) and IL-1β protein expression in Huh-7 and SMMC-7721 cells exposed two different concentrations of luteoloside for 48 h. (B–D) Relative quantitation of NLRP3, Caspase-1 (p10) and IL-1β. (E) A hypothetical cascade pathway of NLRP3 inflammasome suppressed by luteoloside. *, *P*<0.05; **, *P*<0.01; ***, *P*<0.001, versus non-luteoloside-treated control group.

The NLRP3 inflammasome functions as a positive regulator of tumor cells proliferation and metastasis [17], [32]. Activation of the NLRP3 inflammasome is dependent on the generation of ROS [33]. Verifying the inhibitory effect of luteoloside, on the proliferation and metastasis of HCC cells, was accomplished by inhibiting the NLRP3 inflammasome; the levels of NLRP3 inflammasome protein of different treatment groups were determined. The results showed a significant decrease in the expression of NLRP3 of Huh-7 and SMMC-7721 cells treated with luteoloside (25 µM and 50 µM) compared with the non-treated control cells (Fig. 4A, line 1; 4B). Caspase-1 is a family member of intracellular cysteine proteases and they are first synthesized as inactive pro-caspase-1. Upon stimulation pro-caspase-1 zymogen is self activated by proteolytic cleavage into the enzymatically active heterodimer composed of two 10- and 20-kDa subunits. Inflammasome elicits the proteolytic maturation and secretion of interleukin-1β (IL-1β) and IL-18 through caspase-1 activity [17], which was also assessed in luteoloside treated Huh-7 and SMMC-7721 HCC cells. The results from this experiment suggests that luteoloside decreases the proteolytic cleavage of pro-caspase-1 in both Huh-7 and SMMC-7721 HCC cells in a dose-dependent fashion compared with non-treated control cells (Fig. 4A, line 2; 4C). Furthermore, treatment of luteoloside decreased the expression level of IL-1β in both Huh-7 and SMMC-7721 HCC cells (Fig. 4A, line 3; 4D). The results indicate that luteoloside suppresses the proliferation and metastasis of HCC cells by inhibition of NLRP3 inflammasome (Fig. 4E).

### Luteoloside Inhibits *in vivo* Proliferation and Metastasis of HCC Cells

**Figure 5:**
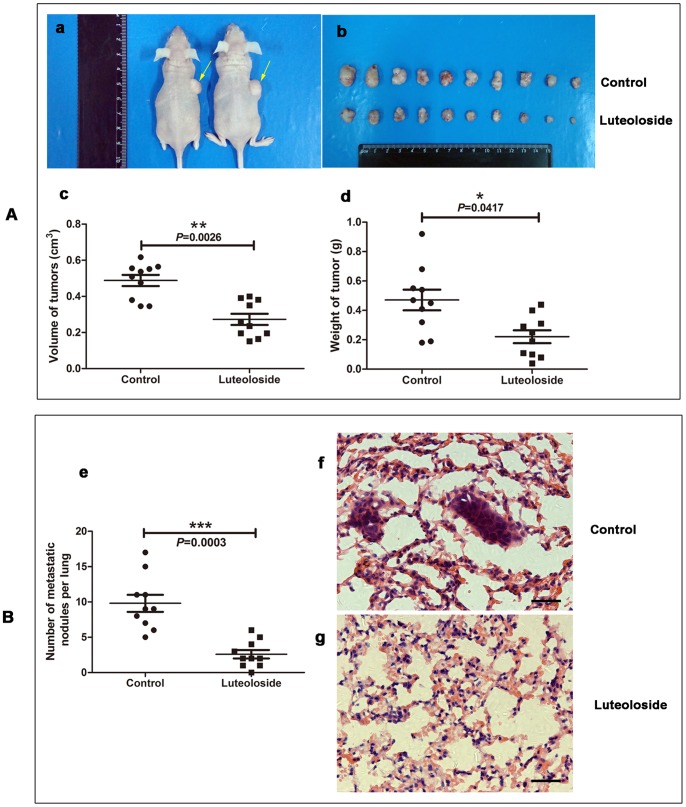
Luteoloside inhibits tumorigenic and spontaneous lung metastatic capabilities of SMMC-7721 cells. (A) Subcutaneous injection of SMMC-7721 cells plus luteoloside treatment in nude mice inhibited tumor growth. (B) Tail vein injection of SMMC-7721 cells plus luteoloside treatment in nude mice inhibited the metastasis of SMMC-7721 cells. (a) 2×10^6^ SMMC-7721 cells were subcutaneously injected into the right upper flank of each mouse. When tumors were observable, the animals were equally divided into two groups (ten per group). The first group received only 0.2 ml of vehicle material by gavage daily and served as a control group. The second group of animals received luteoloside (2 mg/kg body weight) in vehicle, respectively, for 4 weeks. At the termination of the experiment, the mice were sacrificed, and the tumors were weighed immediately after dissection. The yellow arrow shows the tumor. (b) The photo of tumors isolated from killed nude mice of the indicated groups. (c-d) The volume and weight of the tumors. (e) Number of metastatic nodules on the surface of the lungs of mice injected with SMMC-7721 (n = 10 mice in per group) are presented as the means and SEM. (f-g) Representative pictures of lungs with or without metastatic nodules are shown (H&E staining). *, *P*<0.05; **, *P*<0.01; ***, *P*<0.001, versus non-luteoloside-treated control group. Scale bar: 30 µm.

The results obtained from *in vitro* studies showed that treatment of HCC cells with luteoloside inhibits the proliferation, migration and invasion capacity of these cells. To determine the *in vivo* effects of luteoloside, we performed *in vivo* proliferation and metastasis study. The average size and weight of xenografts in the luteoloside-treated group were dramatically smaller and lighter than those of the control group (*P* = 0.0026 and *P* = 0.0417, respectively). (Fig. 5c, 5d). Therefore, the luteoloside treatment significantly inhibited the growth of the xenograft, with inhibition rates (versus the control volume and weight of the tumors) of 44.1 and 53.1%, respectively. Furthermore, we injected SMMC-7721 cells into the lateral tail veins of nude mice (n = 10) and evaluated the metastatic growth of cells in the lung. After 8 weeks, the luteoloside-treated mice displayed a statistically significantly lower number of lung metastases than the control group mice (*P* = 0.0003), indicative of extravasation and tumor growth in the lung (Fig. 5e). When lungs underwent hematoxylin and eosin staining, lung metastases were observed in all ten mice intravenously injected SMCC-7721 cells only, whereas no obvious lung metastases were observed in the mice intravenously injected SMMC-7721 cells with luteoloside treated (Fig. 5f, 5g). It is worth noting that no difference in mouse weight was observed between the treatment group and the control group, suggesting that luteoloside has no adverse effects on mouse growth.

## Discussion

HCC is a rapidly fatal disease, with a life expectancy of about 6 months from the time of the diagnosis. Therapeutic strategies employed to date have significantly improved the prognosis for patients with unresectable HCC. This emphasizes the need for investigating the molecular mechanisms responsible for HCC development and seeking effective and non-cytotoxic chemical agents for chemoprevention and treatment. However, few synthetic antineoplastic compounds have been identified to be effective for the treatment of this disease [3]. In this respect, more and more researchers paid much attention to natural active compounds for cancer chemoprevention and treatment. In the present study, luteoloside, which was previously found to exert antineoplastic effect, was clearly demonstrated to inhibit the proliferation of all six human hepatoma cell lines (Fig. 1B, 1C). In *in vivo* experiments, we obtained the same results (Fig. 5A).

Invasion and metastasis, two of the most important hallmarks of cancer, are the leading lethal factors for malignant cancer, especially for HCC [34]. The long-term survival of HCC patients after curative resection is still confronted by the major obstacle of a high recurrence rate, which is mainly due to the spread of intrahepatic metastases [25]. Therefore, the identification of metastatic factors and an understanding of the underlying molecular pathways that are involved in the progression of metastasis become critical issues. Evidences are accumulating that some flavonoids could significantly inhibit the invasion and metastasis of HCC cells [35], [36]. In this study, luteoloside was shown to dramatically inhibit HCC cell migration, invasion and metastasis both *in vitro* (Fig. 2; Movies S1–S4) and *in vivo* (Fig. 5B).

ROS, such as superoxide (O_2_^−^) and hydrogen peroxide (H_2_O_2_), are constantly produced during metabolic processes in all living species. Under normal physiological conditions, cellular ROS generation is counterbalanced by the action of antioxidant enzymes and other redox molecules. The balance between O_2_^−^ generation and elimination is important for maintaining proper cellular redox states. Recent evident suggests that a moderate increase in ROS can stimulate cell proliferation, invasion and metastasis [37], [38]. However, the precise molecular signaling events of such a regulation are not yet well characterized. In this study, we found that luteoloside could significantly decrease the ROS level of HCC cells, such as Huh-7 and SMMC-7721 cells (Fig. 3).

NLRP3 was recently identified to form a cytoplasmic complex known as the NLRP3 inflammasome, which potently modulates innate immune function by regulating the maturation and secretion of pro-inflammatory cytokines, such as interleukin-1β (IL-1β) [39]. Activation of the NLRP3 inflammasome is dependent on the generation of ROS [40], [41]. In fact, all known NLRP3 activators generate ROS and, conversely, inhibitors of ROS block inflammasome activation [33]. The NLRP3 inflammasome functions as a positive regulator of tumor cells proliferation and metastasis [17], [32], [42]. Several studies have demonstrated that some flavonoids were found to suppress NLRP3 inflammasome activation [43], [44]. Our results showed that luteoloside could significantly decrease the expression of NLRP3 protein of Huh-7 and SMMC-7721 HCC cells (Fig. 4A, lane 1; 4B). Furthermore, luteoloside also decreased the expression level of caspase-1 (p10) (Fig. 4A, lane 2; 4C) and IL-1β (Fig. 4A, lane 3; 4D). Based on the results of the present study, the mechanisms by which luteoloside inhibits HCC cells is summarized in Fig. 4E.

In addition, we found that luteoloside had no significantly effect on the cell apoptosis (Figure S1). Earlier studies have shown that induction of autophagy could result in decreases in mitochondrial ROS generation, NLRP3 protein level, and pro-IL-1β processing [45]. However, in this study, we found that luteoloside had no significantly effect on the protein levels of LC3 and Beclin 1 (Figure S2), two important autophagy markers. The new classification of cell death established by the Nomenclature Committee on Cell Death (NCCD) was based on molecular features [46], [47]. According to this classification, cell deaths can be roughly divided into: apoptosis (caspase dependent extrinsic apoptosis and caspase-independent intrinsic apoptosis), necrosis, autophagy cell death, and other tentative definitions of cell death modalities including anoikis, entosis, pyroptosis, netosis and cornification. In HCC, at least four types of cell death pathways have been observed and studied, including apoptosis [48], necrosis [49], autophagy [50], anoikis [51], None of the above described cell deaths contribute to HCC proliferation and metastasis equally, and HCC progression is not dependent entirely on any single cell death pathway. In this study, we found that luteoloside had no significantly effects on the cell apoptosis or autophagy. Further study is underway to explore whether luteoloside has significantly effects on other kinds of cell death.

Luteoloside significantly inhibited the proliferation of HCC cells *in vitro* and *in vivo*. But, luteoloside had no significantly effect on the cell apoptosis or autophagy. Jørgensen *et al* have shown that the predominant effect of nilotinib, a kind of tyrosine kinase inhibitor, is antiproliferative rather than proapoptotic. They further suggested that combining nilotinib with other drugs should be carefully considered from the point of view of merely inducing G_0_/G_1_ block without apoptosis [52]. Papeleu *et al* found Trichostatin A, a drug candidate for cancer therapy, could inhibit cell proliferation at different steps of the cell cycle. But they also found Trichostatin A did not induce apoptosis in cells. Their finding supports its use in the treatment of proliferative disorders [53]. So, from another perspective, perhaps the predominant effect of luteoloside is antiproliferative rather than an “executor” of cell death. Further studies are required to explore this possibility.

To the best of our knowledge, this is the first to show that luteoloside, a flavone subclass of flavonoids, inhibits the proliferation, invasion and metastasis of HCC cells through inhibition of NLRP3 inflammasome. Our findings provide an important basis for a further exploration towards understanding the action mechanisms of luteoloside and possibly its beneficial effect in the prevention of tumor proliferation, invasion and metastasis.

## Supporting Information

Figure S1 **Luteoloside does not affect the apoptosis rate of Huh-7 and SMMC-7721 cells.** (A–B) The effect of luteoloside on caspase activity. Cells were plated in a 96-well plate. Overnight, the cells were incubated with different concentrations of luteoloside. After 24 hours, caspase-3/7 activity was measured using the Caspase-Glo® 3/7 Assay (Promega, Madison, WI). The caspase-3/7 activity was proportionate to the produced luminescence intensity. (C) Detection of DNA ladder formation in Huh-7 and SMMC-7721 cells after treatment with luteoloside for 24 hours. (D) Hoechst 33342 staining. The cells treated with luteoloside and stained with Hoechst 33342. Arrows show apoptotic small bodies. NS, not significant (*P*>0.05). Scale bars: 1 µm.
(TIF)

Figure S2 **Luteoloside does not affect autophagy.** Western blot analyses of LC3 and Beclin 1 protein expression in Huh-7 and SMMC-7721 cells exposed two different concentrations of luteoloside for 48 h.
(TIF)

Movie S1 **The migration of non-luteoloside-treated SMMC-7721 cells into the wound was monitored using a CellVoyager CV1000 confocal scanner system.** The images were acquired every hour for 72 hours.
(MP4)

Movie S2 **The migration of luteoloside-treated SMMC-7721 cells into the wound was monitored using a CellVoyager CV1000 confocal scanner system.** The images were acquired every hour for 72 hours.
(MP4)

Movie S3 **The migration of non-luteoloside-treated Huh-7 cells into the wound was monitored using a CellVoyager CV1000 confocal scanner system.** The images were acquired every 0.5 hour for 48 hours.
(MP4)

Movie S4 **The migration of luteoloside-treated Huh-7 cells into the wound was monitored using a CellVoyager CV1000 confocal scanner system.** The images were acquired every 0.5 hour for 48 hours.
(MP4)
