# Supplementary figures and images for: Luteoloside Suppresses Proliferation and Metastasis of Hepatocellular Carcinoma Cells by Inhibition of NLRP3 Inflammasome
Source: PLoS One. 2014 Feb 26;9(2):e89961. doi: 10.1371/journal.pone.0089961 (PMC3935965; doi:10.1371/journal.pone.0089961)

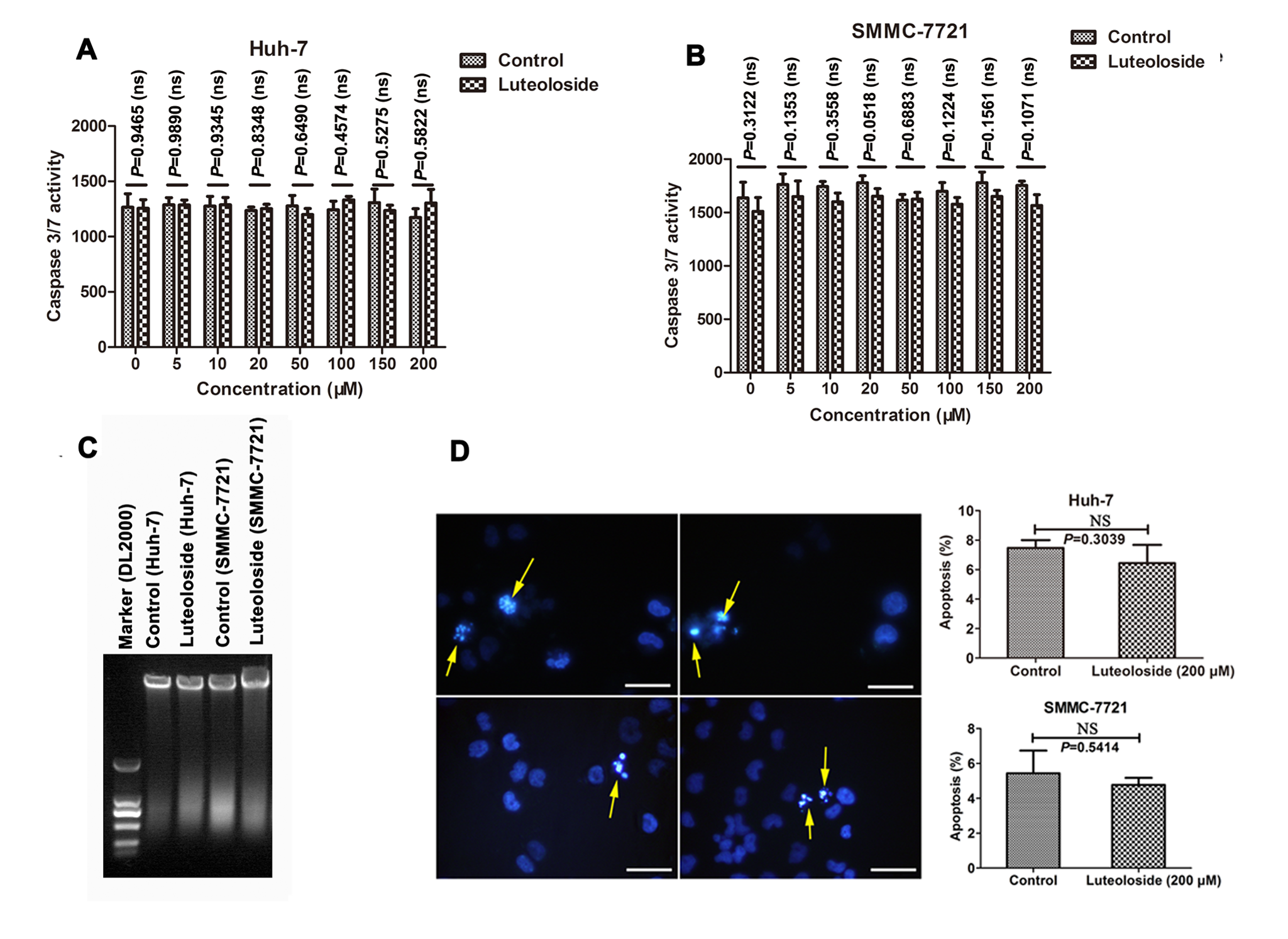

Supplement: Figure S1 — Luteoloside does not affect the apoptosis rate of Huh-7 and SMMC-7721 cells. (A–B) The effect of luteoloside on caspase activity. Cells were plated in a 96-well plate. Overnight, the cells were incubated with different concentrations of luteoloside. After 24 hours, caspase-3/7 activity was measured using the Caspase-Glo® 3/7 Assay (Promega, Madison, WI). The caspase-3/7 activity was proportionate to the produced luminescence intensity. (C) Detection of DNA ladder formation in Huh-7 and SMMC-7721 cells after treatment with luteoloside for 24 hours. (D) Hoechst 33342 staining. The cells treated with luteoloside and stained with Hoechst 33342. Arrows show apoptotic small bodies. NS, not significant (P>0.05). Scale bars: 1 µm. (TIF) [file pone.0089961.s001.tif]

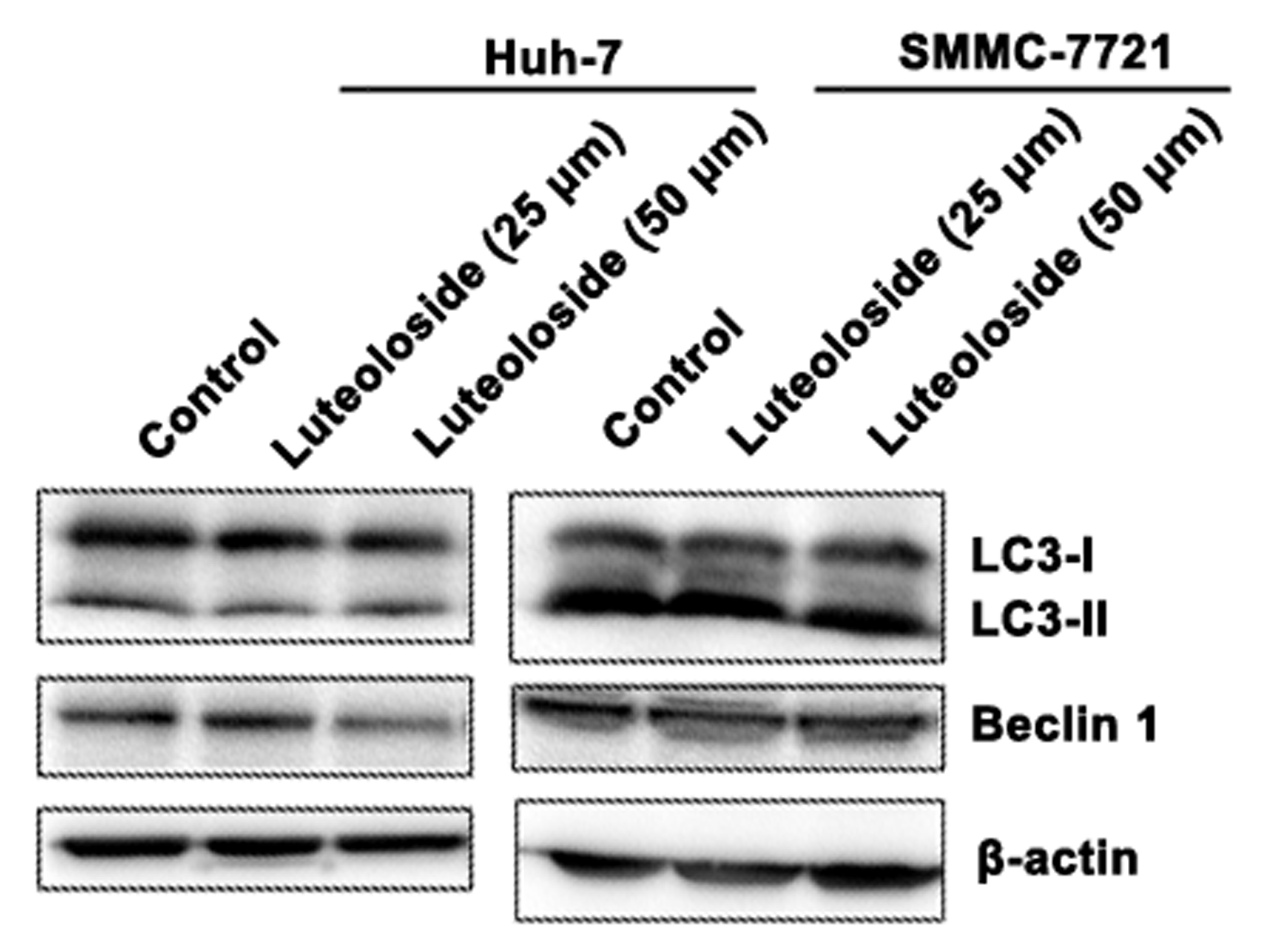

Supplement: Figure S2 — Luteoloside does not affect autophagy. Western blot analyses of LC3 and Beclin 1 protein expression in Huh-7 and SMMC-7721 cells exposed two different concentrations of luteoloside for 48 h. (TIF) [file pone.0089961.s002.tif]
